# Inhibition of STAT3^Y705^ phosphorylation by Stattic suppresses proliferation and induces mitochondrial-dependent apoptosis in pancreatic cancer cells

**DOI:** 10.1038/s41420-022-00922-9

**Published:** 2022-03-14

**Authors:** Hangcheng Guo, Yanyi Xiao, Ziwei Yuan, Xuejia Yang, Jiawei Chen, Chaoyue Chen, Mengsi Wang, Lili Xie, Qinbo Chen, Yu Tong, Qiyu Zhang, Yongheng Bai

**Affiliations:** 1 Key Laboratory of Diagnosis and Treatment of Severe Hepato-Pancreatic Diseases of Zhejiang Province, The First Affiliated Hospital of Wenzhou Medical University, Wenzhou, 325000 China; 2 Department of Laboratory Medicine, People’s Hospital of Wenzhou City, Wenzhou, 325000 China; 3 Department of Hepato-Biliary-Pancreatic Surgery, The First Affiliated Hospital of Wenzhou Medical University, Wenzhou, 325000 China; 4 Health Assessment Center, Wenzhou Medical University, Wenzhou, 325000 China

**Keywords:** Pancreatic cancer, Targeted therapies, Drug screening

## Abstract

Patients with pancreatic cancer (PC) show dismal prognosis and high mortality. The development of PC is associated with the overactivation of STAT3. Here, we have determined that the non-peptide small molecule Stattic inhibits PC development by targeting STAT3. In vitro, Stattic treatment time- and dose-dependently inhibited proliferation of pancreatic cancer cells (PCCs) by reducing c-Myc expression and enhancing p53 activity. Consequently, p-Rb, cyclin D1, Chk1, and p21 (cell cycle proteins) were downregulated, and PCCs were arrested at the G1 phase, which was also confirmed by decreased Ki67 expression and unaltered PCNA expression. In addition, Stattic-induced mitochondrial-dependent apoptosis by elevating cleaved caspase-3, and Bax, cytochrome C levels, while reducing expression of Bcl-2, which may be regulated by reduced survivin expression. Further studies showed that Stattic exerts its anti-tumor effect via inhibition of STAT3^Y705^ phosphorylation and nuclear localization in PCCs. In a nude mouse tumorigenesis model, Stattic inhibited PC growth by antagonizing STAT3^Y705^ phosphorylation. Interleukin-6 used as a molecule agonist to activate STAT3, as well as overexpression of STAT3, could partially reverse Stattic-mediated anti-proliferation and pro-apoptotic effects of PCCs. Thus, these findings indicate that inhibition of STAT3^Y705^ phosphorylation by Stattic suppresses PCC proliferation and promotes mitochondrial-mediated apoptosis.

## Introduction

Pancreatic cancer (PC) is generally associated with poor prognosis. By 2030, PC is projected to rank as the second leading killer of cancer in the United States [1–3]. Although surgical resection is widely used for PC management, long-term prognosis of PC patients remains very poor [4]. Most PC patients receive chemotherapy as mainstay treatment, but resistance to chemotherapy drugs leads to poor treatment effects [5, 6]. For this reason, new and more effective drugs are necessary.

Impaired apoptosis and proliferation of PCCs are regarded as a major cause of poor prognosis in PC [7]. The JAK2/STAT3 transduction axis modulates apoptosis of cancer cells. Extensive evidence shows that STAT3 overactivation induces angiogenesis, immunosuppression, and metastasis, and suppresses apoptosis and inflammation, eventually resulting in PC among other cancers [8–10]. After JAK2 is activated by IL-6 or other inducers, the Src homology domain 2 (SH2) of STAT3 binds to the corresponding site of phosphotyrosine of JAK2, which then causes STAT3 phosphorylation at Tyr705 (Y705). STAT3^Y705^ phosphorylation can induce itself to form dimers and is incorporated into the nuclear expression [11, 12]. As a result, activated STAT3 upregulates proliferation-related factors, leading to excessive proliferation of tumor cells. In addition, STAT3 upregulates the expression of certain proteins that prevent apoptosis, and suppresses proteins responsible for apoptosis activation. Moreover, STAT3 alters cell cycle progression by targeting cell cycle-related regulatory factors [12, 13]. In PC, JAK2/STAT3 signaling is usually abnormally activated. Enhanced STAT3 activity promotes PCC malignance and is strongly associated with poor prognosis [14–16]. Thus, targeting STAT3 could potentially be utilized as effective anti-PC strategies.

Specific small molecule inhibitors have been developed to target STAT3, although no drugs have been used in the clinic. The identification and development of new drugs that activate STAT3 remain an important scientific and clinical challenge [17, 18]. Stattic is a non-peptide small molecule inhibitor that selectively inhibits STAT3 activity by binding to the SH2 region. The SH2 region is essential for the Y705 phosphorylation and nuclear translocation of STAT3 [19, 20]. Based on the aforementioned concepts, we postulated that Stattic can prevent PC by decreasing phosphorylation of STAT3^Y705^.

Herein, the anti-tumor effect of Stattic on PCC proliferation, apoptosis, ferroptosis, and cell cycle was explored in vitro and using a nude mouse tumorigenesis model in vivo. In addition, the activity of JAK2/STAT3 signaling was also evaluated to explore the underlying mechanism of Stattic. Our findings identified an anti-tumor activity of Stattic by suppressing STAT3^Y705^ phosphorylation, indicating its potential of Stattic as an intervention for PC.

## Results

### Stattic induces G1 arrest to inhibit PCC proliferation via suppression of c-Myc level and upregulation of p53 activity

**Fig. 1:**
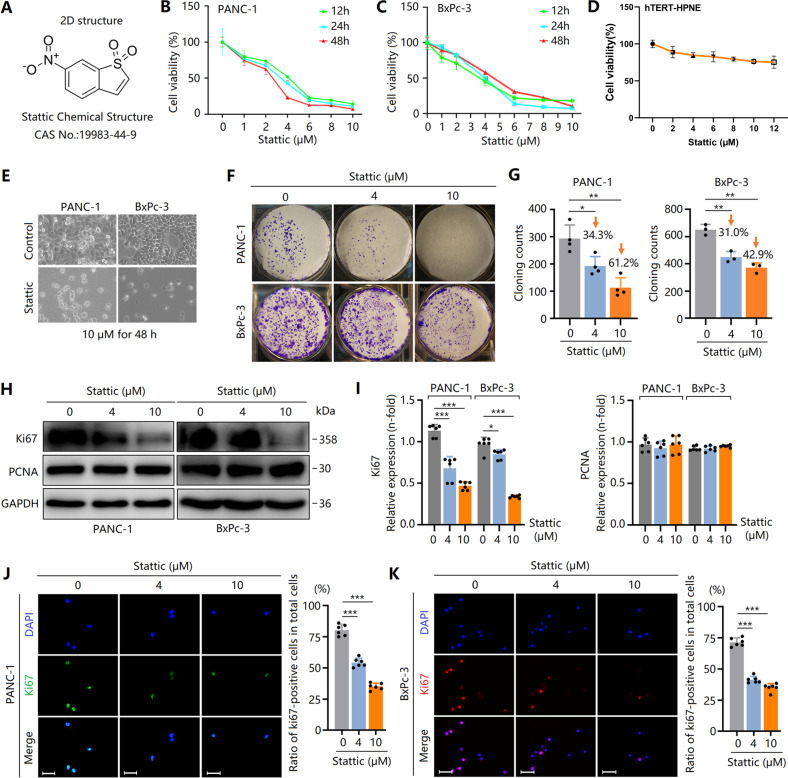
Stattic time- and dose-dependently inhibits proliferation of PCCs. PCCs were treated with various concentrations of Stattic for 24 h or 48 h. **A** Chemical structure of Stattic. **B**–**D** Viability of PANC-1, BxPc-3, and hTERT-HPNE cells with or without Stattic treatment (0–12 μM) analyzed by CCK-8 assay. **E** Morphology of PANC-1 and BxPc-3 cells treated with 10 μM Stattic for 48 h. **F**, **G** The proliferation of PANC-1 and BxPc-3 cells with or without Stattic analyzed by colony formation assay. **H**, **I** Expression of PCNA and Ki67 in Stattic-treated BxPc-3 and PANC-1 cells. **J**, **K** Immunocytochemical staining of Ki67 in Stattic-treated BxPc-3 and PANC-1 cells. Bar = 50 μm. *P < 0.05, **P < 0.01, ***P < 0.001.

Results shown in Fig. 1B and C reveal that Stattic decreased PANC-1 and BxPc-3 cell proliferation in a concentration- and time-dependent manner. We also observed that the proliferation of hTERT-HPNE cells did not change after treatment of Stattic (Fig. 1D), suggesting that Stattic controls PCCs but not affect PDECs proliferation. The IC_50_ value of BxPc-3 and PANC-1 cells following Stattic treatment for 24 h were 3.135–5.296 μM and 3.835–4.165 μM, respectively. The Stattic (10 μM)-induced anti-proliferative effects in PCCs were also confirmed by the reduced number of adherent cells observed under the microscope (Fig. 1E). In addition, Stattic treatment resulted in a reduction in the number of colony formation of PCCs (Fig. 1F, G). Thus, Stattic imparted markedly anti-proliferative effects on PCCs.

**Fig. 2:**
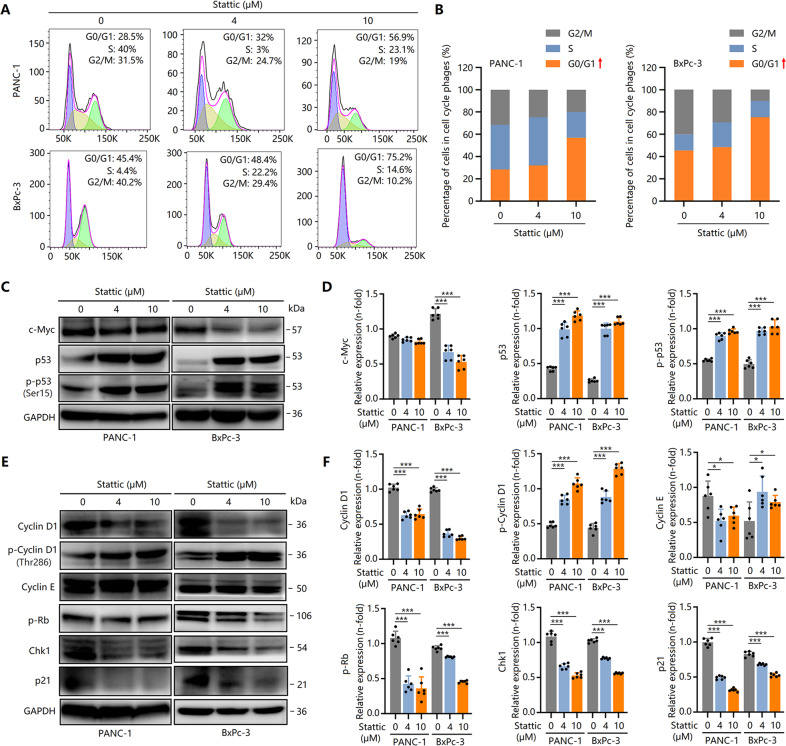
Stattic induces cell cycle G1 arrest by downregulating c-Myc expression and upregulating p53 activity. PCCs were treated with 4 and 10 μM of Stattic for 24 h. **A**, **B** Flow cytometry analysis for cell cycle in Stattic-treated PANC-1 and BxPc-3 cells. **C**, **D** Western blot analysis showing the expression of c-Myc and p53, and the phosphorylation of p53 in Stattic-treated PANC-1 and BxPc-3 cells. **E**, **F** Western blot analysis showing the expression of cyclin D1, cyclin E, Chk1, and p21 and the phosphorylation of cyclin D1 and Rb in Stattic-treated BxPc-3 and PANC-1 cells. *P < 0.05, ***P < 0.001.

Further studies revealed that Ki67 expression was dose-dependently downregulated by Stattic in PCCs (Fig. 1H–K). However, interestingly, the expression of PCNA, another marker for cellular proliferation, did not result in a marked decrease (Fig. 1H, I). Ki67 expression has been demonstrated to alter the cell cycle [21]. Ki67 was expressed in G1 (late stage), S, G2, and M phases, but did not in the G1 (early stage) and G0 phases. However, PCNA was mainly expressed in the early stage of the G1 phase, and Ki67 was not expressed at this time, indicating that G1 arrest contributes to Stattic-induced suppression of PCC proliferation. To verify this, a flow cytometry test showed that Stattic-treated PCCs were enriched in the G1 phase (Fig. 2A, B). Thus, Stattic time- and dose-dependently inhibited PCC proliferation via G1 arrest.

To further clarify the anti-proliferative mechanism of Stattic, we investigated the role of c-Myc and p53, which are regarded as important inducers that trigger cycle arrest [21, 22], in Stattic-treated PCCs. Stattic reduced c-Myc expression in BxPc-3 cells, but not in PANC-1 cells (Fig. 2C, D; Fig. S1A). In addition, Stattic enhanced the gene and protein expression of p53 in PCCs (Fig. 2C, D; Fig. S1B). Moreover, the phosphorylation of p53 was also induced by Stattic (Fig. 2C, D). Thus, downregulated c-Myc levels and elevated p53 activity are involved in Stattic-mediated anti-proliferative effects. These findings were also confirmed by the reduced levels of cell cycle-associated regulatory factors, including cyclin D1, p-Rb, Chk1, and p21 (Fig. 2E, F; Fig. S1C), which are regulated by c-Myc and p53.

Collectively, Stattic downregulates c-Myc levels and upregulates p53 activity, consequently triggering G1 phase arrest, and ultimately inhibiting PCC proliferation.

### Stattic induces mitochondrial-dependent apoptosis in PCCs in different ways

**Fig. 3:**
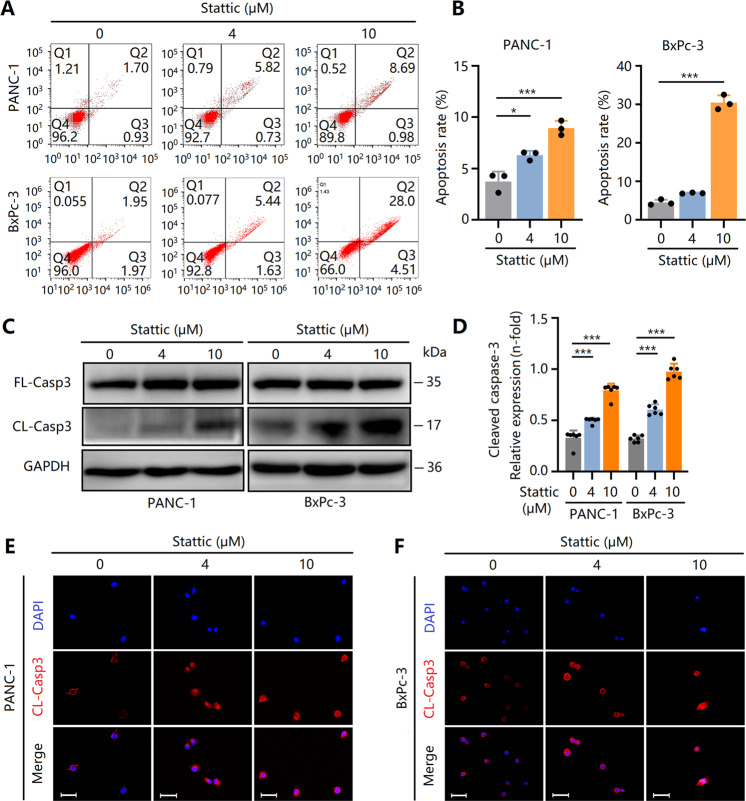
Stattic induces apoptosis of PCCs via a cleaved-caspase-3-dependent mechanism. PCCs were treated with 4 and 10 μM of Stattic for 24 h. **A**, **B** Apoptosis of Stattic-treated BxPc-3 and PANC-1 cells. **C**, **D** Expression of full-length caspase-3 (FL-Casp3) and cleaved caspase-3 (CL-Casp3) in Stattic-treated PANC-1 and BxPc-3 cells. **E**, **F** Immunocytochemical staining of cleaved caspase-3 in Stattic-treated PANC-1 and BxPc-3 cells. Bar = 50 μm. *P < 0.05, ***P < 0.001.

We previously showed that Stattic induces G1 arrest in PCCs; however, we wanted to determine whether cellular apoptosis also occurs in Stattic-treated PCCs. Flow cytometry analysis revealed that Stattic upregulated apoptosis, particularly in BxPc-3 cells (Fig. 3A, B). Full-length caspase-3 (FL-Casp3) is one of the terminal apoptosis-inducing proteins, and its spliceosome cleaved caspase-3 (CL-Casp3) is an active protein with executive functions [23]. Of note, CL-Casp3 expression was dose-dependently elevated in Stattic-treated PCCs (Fig. 3C–F), indicating that Stattic treatment triggered PCC apoptosis.

**Fig. 4:**
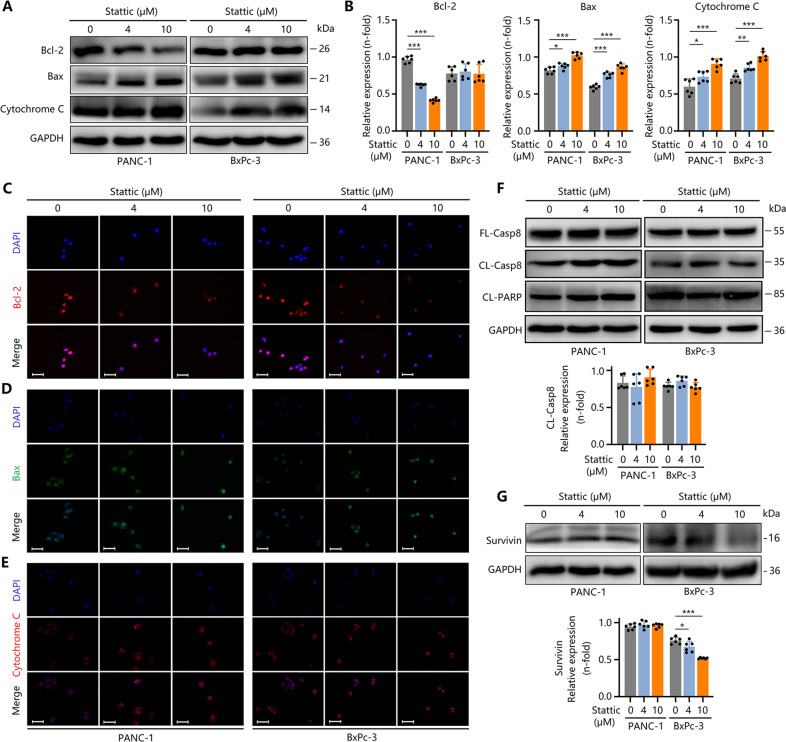
Stattic induces apoptosis of PCCs via a mitochondrial-dependent pathway. PCCs were treated with 4 and 10 μM of Stattic for 24 h. **A**, **B** Expression of Bax, Bcl-2, and cytochrome C in Stattic-treated PANC-1 and BxPc-3 cells. **C**–**E** Immunocytochemical staining for Bcl-2, Bax, and cytochrome C in Stattic-treated PANC-1 and BxPc-3 cells. Bar = 50 μm. **F** Expression of cleaved PARP (CL-PARP), cleaved caspase-8 (CL-Casp8), and full-length caspase-8 (FL-Casp8). **G** Expression of survivin in Stattic-treated BxPc-3 and PANC-1 cells. *P < 0.05, **P < 0.01, ***P < 0.001.

Next, we investigated the mechanism of apoptosis in Stattic-treated PCCs. In PANC-1 cells, Bax and cytochrome C expression levels were upregulated, and Bcl-2 expression was downregulated by Stattic (Fig. 4A–E). Although in BxPc-3 cells, Bcl-2 expression was not decreased by Stattic treatment, the expression of Bax and cytochrome C increased (Fig. 4A–E). These results indicated that Stattic-triggered apoptosis involves the mitochondrial-dependent pathway. Furthermore, Stattic did not affect the expression of CL-Casp8 and cleaved-PARP (Fig. 4F), suggesting that Stattic-induced apoptosis does not occur via the death receptor pathway. In addition to the anti-apoptotic protein Bcl-2, another important anti-apoptotic protein, survivin, may be involved in PCC apoptosis induced by Stattic. The expression of survivin in BxPc-3 cells, but not in PANC-1 cells, was markedly downregulated after treatment with Stattic (Fig. 4G). Combined with the aforementioned observations, we hypothesized that in different PCCs, Stattic-triggered apoptosis may act in different ways. Stattic-induced apoptosis in PANC-1 cells by inhibiting Bcl-2 expression, whereas in BxPc-3 cells, this was triggered by suppression of survivin expression.

In addition to apoptosis, ferroptosis has been reported as a potential new approach for anti-tumor therapy [24, 25]. Therefore, we investigated whether Stattic induced PCC ferroptosis. First, oxidative stress was evaluated in Stattic-treated PCCs. The levels of MDA did not change in PCCs after Stattic treatment (Fig. S2A). Also, Stattic did not affect GSH-Px activity (Fig. S2B). These findings indicated that Stattic did not induce oxidative stress injury. Second, the expression of GPX4, a highly specific negative marker of ferroptosis, was analyzed. We found that the expression of GPX4 in PANC-1 and BxPc-3 cells were both lower than in hTERT-HPNE cells (Fig. S2C), indicating that there was a certain degree of ferroptosis, which may be a self-adaptive response of tumor cells. Finally, we analyzed the expression of GPX4 and xCT (another ferroptosis negative marker). Stattic did not downregulate GPX4 and xCT expression in PANC-1 cells (Fig. S2D). However, interestingly, the expression of GPX4 and xCT in BxPc-3 cells was reduced (Fig. S2D), suggesting that Stattic may induce ferroptosis of BxPc-3 cells. We speculated that PCC genetic background, including *KRAS* mutations, may play an important role in ferroptosis, but the specific mechanism requires further investigation.

### Stattic selectively antagonizes STAT3^Y705^ phosphorylation and inactivates STAT3 in PCCs

**Fig. 5:**
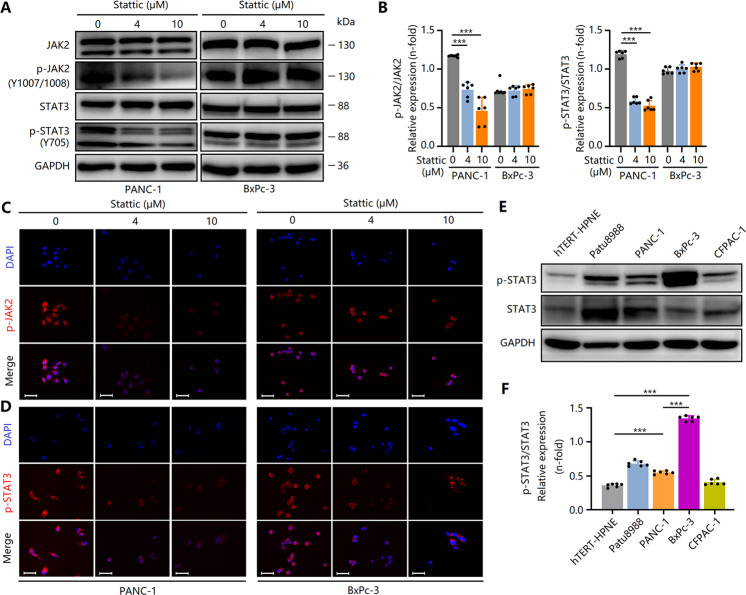
Stattic selectively antagonizes STAT3^Y705^ phosphorylation and inactivates STAT3 in PCCs. PCCs were treated with 4 and 10 μM of Stattic for 24 h. **A**, **B** Expression and phosphorylation of JAK2 and STAT3 in Stattic-treated BxPc-3 and PANC-1 cells. **C**, **D** Immunocytochemical staining of p-JAK2 and p-STAT3 in Stattic-treated PANC-1 and BxPc-3 cells. Bar = 50 μm. **E**, **F** Expression and phosphorylation of STAT3 in different PCCs. Data are shown as the mean ± standard deviation. ***P < 0.001.

To further explore the potential anti-tumor mechanisms of Stattic, we evaluated the effects of Stattic on the activities of several signaling pathways that are involved in the occurrence and development of PC. In PCCs, Stattic did not change the phosphorylation (S675) of β-catenin (Fig. S3A, B). In addition, Stattic did not downregulate mTOR activity (Fig. S3C, D). Moreover, the expression of Gli1 and Gli2 were also not affected by Stattic (Fig. S3E, F). A previous study has shown that Stattic is a selective inhibitor that antagonizes STAT3 activity by binding to the SH2 region [26]. Thus, we also evaluated the effect of Stattic on STAT3 expression and phosphorylation, as well as JAK2 activity. As expected, the phosphorylation of JAK2 (Y1007 + Y1008) and STAT3 (Y705) in PANC-1 cells was downregulated by Stattic treatment (Fig. 5A–D). Interestingly, Stattic did not inhibit the phosphorylation of JAK2 and STAT3 in BxPc-3 cells (Fig. 5A–D). The phosphorylation level of STAT3 in BxPc-3 cells was higher than that in PANC-1 cells (Fig. 5E, F). We speculated that the effect of Stattic was not obvious at the concentration range of 0–10 μM due to the high activity of STAT3. In addition, differences in the genetic background of different PCC lines, including KRAS and SMAD4 mutations, alters the effect of Stattic on STAT3 activity. Thus, Stattic selectively antagonized STAT3^Y705^ phosphorylation and decreased STAT3 activity in specific PCC.

### Stattic inhibits PC growth in a nude mouse tumor model by inactivating STAT3

**Fig. 6:**
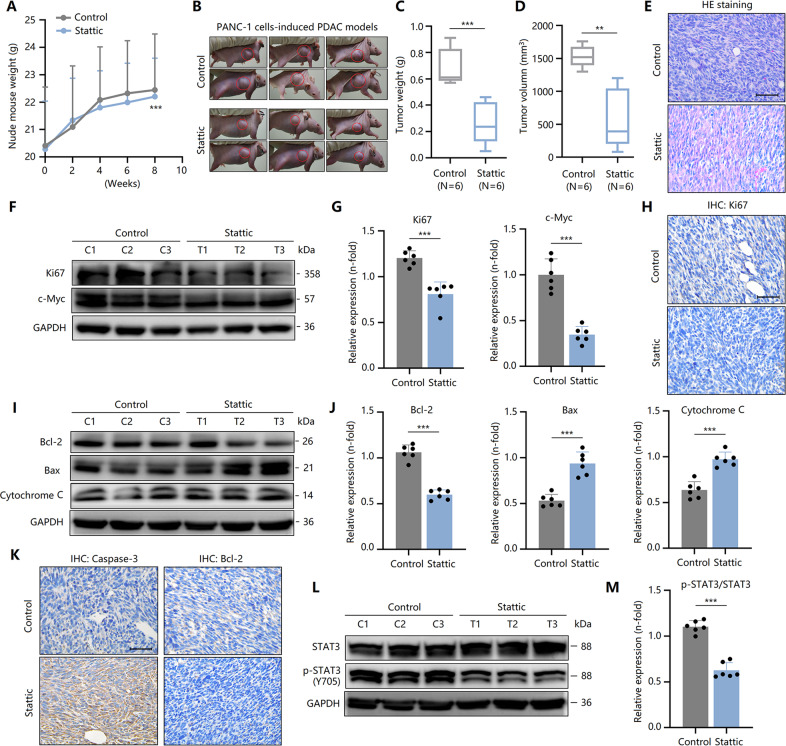
Stattic inhibits pancreatic cancer growth in a nude mouse tumor model by inducing STAT3 inactivation. PANC-1 cells (5 × 10^6^ cells) in 100 μL of PBS were injected subcutaneously into the abdominal side of experimental mice (*n* = 12), and then the mice (*n* = 6) were intragastrically administered Stattic (10 mg/kg) daily for 30 days. **A** The body weights of nude mice measured at different time points. **B** Pancreatic tumor xenograft in vivo models established by subcutaneous implantation of PANC-1 cells. **C** Tumor weights of nude mice measured after Stattic treatment. **D** Tumor volume of nude mice measured after Stattic treatment. **E** HE staining in Stattic-treated pancreatic cancer tissues. Bar = 100 μm. **F**, **G** The expression of Ki67 and c-Myc in Stattic-treated pancreatic cancer tissues. **H** IHC staining of Ki67 in Stattic-treated pancreatic cancer tissues. Bar = 100 μm. **I**, **J** Expression of Bcl-2, Bax, and cytochrome C in Stattic-treated pancreatic cancer tissues. **K** IHC staining of caspase-3 and Bcl-2 in Stattic-treated pancreatic cancer tissues. Bar = 100 μm. **L**, **M** Expression and phosphorylation of STAT3 in Stattic-treated pancreatic cancer tissues. Data are shown as the mean ± standard deviation. **P < 0.01, ***P < 0.001.

As mentioned above, our in vitro experiments revealed the anti-tumor activity of Stattic in PCCs. Here, we further clarified the effect of Stattic on PC growth by constructing a nude mouse tumorigenesis model in vivo. After injecting PANC-1 cells subcutaneously into the neck of nude mice, the weight of nude mice significantly increased over time (Fig. 6A). After the tumor had developed, the nude mice received daily injection consecutively for 4 weeks with Stattic treatment. As a result, the weight and volume of the tumor significantly decreased (Fig. 6C, D). HE staining clarified the morphological characteristics of the tumor tissue (Fig. 6E). Meanwhile, other tissues in nude mice, including the liver, lung, and kidney, were evaluated and confirmed that this particular Stattic concentration does not induce toxicity (Fig. S4). Stattic treatment markedly decreased the expression of Ki67 and c-Myc (Fig. 6F–H), indicating the inhibitory effect of Stattic on PCC proliferation. In addition, downregulated expression of Bcl-2 and upregulated expression of Bax and cytochrome C were observed in Stattic-treated PC tissues (Fig. 6I–K), suggesting that mitochondrial-dependent apoptosis of PCCs was induced by Stattic. Mechanistically, the inhibition of STAT3^Y705^ phosphorylation in tumor tissues confirmed again anti-tumorigenic effects of Stattic by antagonizing STAT3 activity (Fig. 6L, M). Collectively, these in vivo findings show that Stattic suppressed PC growth in a nude mouse tumor model by inactivating STAT3.

### IL-6 partially abolishes the inhibitory effect of Stattic in PCCs via STAT3

**Fig. 7:**
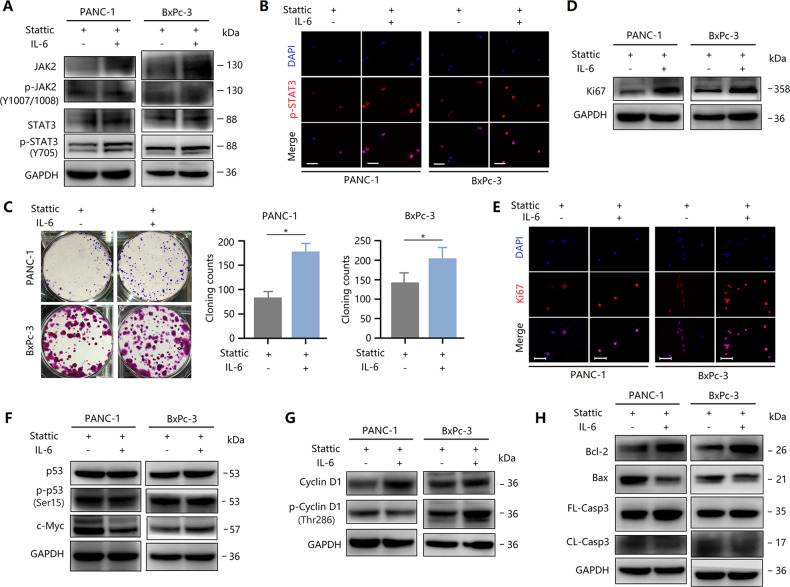
IL-6 partially abolishes the inhibitory effect of Stattic in PCCs via STAT3. PCCs were treated with 10 μM of Stattic with or without IL-6 for 24 h. **A** Expression and phosphorylation of STAT3 and JAK2 in the indicated groups. **B** Immunocytochemical staining of p-STAT3 in Stattic-treated PANC-1 and BxPc-3 cells with or without IL-6. Bar = 50 μm. **C** Colony formation assay revealing the proliferation of Stattic-treated PANC-1 and BxPc-3 cells with or without IL-6. **D** Expression of Ki67 in Stattic-treated cells with or without IL-6. **E** Immunocytochemical staining of Ki67 in Stattic-treated PANC-1 and BxPc-3 cells with or without IL-6. Bar = 50 μm. **F** Expression of c-Myc and p53 activity in cells treated with or without IL-6. **G** Expression and phosphorylation of cyclin D1 in Stattic-treated PANC-1 and BxPc-3 cells with or without IL-6. **H** Western blot analysis showing the expression of Bcl-2, Bax, FL-Casp3, and CL-Casp3 in Stattic-treated PANC-1 and BxPc-3 cells with or without IL-6. *P < 0.05.

IL-6 is an important JAK2/STAT3 signaling inducer and is involved in PC development [13, 17]. Here, we investigated whether IL-6-induced STAT3 activation can reverse the anti-tumor effect of Stattic in PCCs. First, the JAK2/STAT3 activity was evaluated after treatment with recombinant IL-6. As expected, IL-6 enhanced the phosphorylation levels of JAK2 (Y1007 + Y1008) and STAT3 (Y705) in Stattic-treated PCCs (Fig. 7A). Also, IL-6 partially abolished the inhibitory effect of Stattic on nuclear expression of phosphorylated STAT3 (Fig. 7B). These results indicated that IL-6 antagonized to a certain extent the decrease of STAT3 activity caused by Stattic. As a result, Stattic-induced reduction in clone formation of PCCs was alleviated by IL-6 (Fig. 7C). Further studies revealed that IL-6 restored Stattic-induced downregulation of Ki67 expression (Fig. 7D, E). However, surprisingly, IL-6 did not reverse p53 activity in Stattic-treated PANC-1 cells (Fig. 7F). Furthermore, c-Myc expression was not reversed by IL-6 (Fig. 7F), suggesting that there were other mechanisms for IL-6 to abolish the proliferation inhibition caused by Stattic in PANC-1 cells. Although the Stattic-mediated decrease in p53 activity in BxPc-3 cells was not reversed by IL-6 (Fig. 7F), Stattic-induced reduction in c-Myc expression was restored by IL-6 (Fig. 7F), thereby promoting the phosphorylation of cyclin D1 (Fig. 7G). In addition, IL-6 partially abolished the apoptosis-inducing effect of Stattic, including the downregulated expression of CL-Casp3 and Bax and upregulated expression of Bcl-2 (Fig. 7H). Taken together, IL-6 partially abolished the inhibitory effect of Stattic in PCCs by reactivating STAT3.

### Lentivirus-mediated overexpression of STAT3 partially reverses the anti-tumor effect of Stattic

**Fig. 8:**
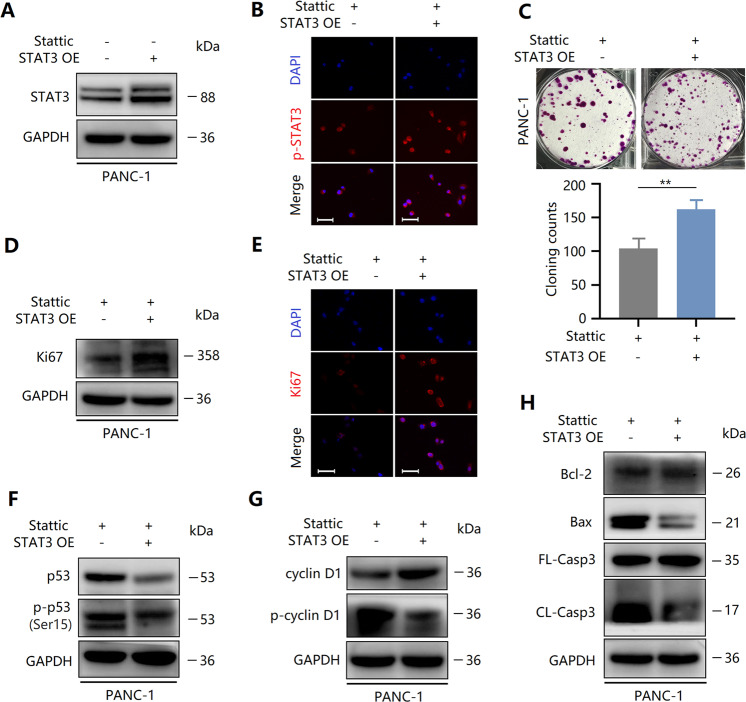
Lentivirus-mediated overexpression of STAT3 partially reverses the anti-tumor effect of Stattic. **A** Expression of STAT3 without Stattic-treated PANC-1 cells after transfection with lentivirus. **B** Immunocytochemical staining of p-STAT3 in Stattic-treated PANC-1 cells after transfection with lentivirus. Bar = 50 μm. **C** Colony formation assay revealing the proliferation of Stattic-treated PANC-1 cells after transfection with lentivirus. **D** The expression of Ki67 in Stattic-treated PANC-1 cells after transfection with lentivirus. **E** Immunocytochemical staining of Ki67 in Stattic-treated PANC-1 cells after transfection with lentivirus. Bar = 50 μm. **F** Expression and phosphorylation of p53 in Stattic-treated PANC-1 cells after transfection with lentivirus. **G** Expression and phosphorylation of cyclin D1 in Stattic-treated PANC-1 cells after transfection with lentivirus. **H** Proteins levels of Bcl-2, CL-Casp3, Bax, and FL-Casp3 in PANC-1 cells subjected to the indicated treatments. **P < 0.01.

As mentioned above, STAT3 activity in PANC-1 cells was lower than that in BxPc-3 cells (Fig. 5E and F). Thus, STAT3 overexpression was induced in PANC-1 cells using lentiviruses to reevaluate the effect of activated STAT3 on the anti-tumor effect of Stattic. Our results confirmed the overexpression of STAT3 protein in PANC-1 cells (Fig. S5A and B). In addition, without Stattic-treated PANC-1 cells, STAT3 expression was also enhanced (Fig. 8A), and thereby phosphorylated STAT3 was induced to enter the nucleus and reactivated (Fig. 8B). Similar to IL-6, STAT3 overexpression alleviated Stattic-induced reduction in clone formation of PANC-1 cells (Fig. 8C), which might be associated with the abolishment of Stattic-triggered Ki67 inhibition (Fig. 8D, E). Further studies showed that discordant to the effects of IL-6, enhanced p53 expression and phosphorylation in Stattic-treated PANC-1 cells were partially reversed (Fig. 8F), suggesting that STAT3 and p53 may have a more direct relationship than IL-6. As a result, the activity of cyclin D1 was restored by STAT3 overexpression to re-participate in the regulation of the cell cycle (Fig. 8G). In addition, the Stattic-induced increase in apoptosis of PANC-1 cells was also abolished by STAT3 overexpression (Fig. 8H). Thus, lentivirus-mediated overexpression of STAT3 partially reversed the anti-tumor effect of Stattic via STAT3 reactivation.

As mentioned above, Stattic may be involved in ferroptosis of BxPc-3 cells (Fig. S2D). In PANC-1 cells, ferroptosis was not induced by Stattic treatment, as determined by GPX4 and xCT expression. Previous studies have shown that STAT3 is a positive regulator of ferroptosis, and abnormal activation of STAT3 in PCCs can promote ferroptosis by inducing cathepsin B expression and lysosomal membrane permeabilization [27, 28]. Thus, we also evaluated the effects of IL-6-induced STAT3 activation in PANC-1 and BxPc-3 cells or lentivirus-mediated STAT3 overexpression in PANC-1 cells on ferroptosis. First, IL-6 treatment did not affect the level of MDA and the activity of GSH-Px in Stattic-treated PCCs (Fig. S6A, B). Second, IL-6 reversed Stattic-induced reduction in GPX4 and xCT expression in BxPc-3 cells, although IL-6 did not affect GPX4 and xCT expression in PANC-1 cells (Fig. S6C). Third, MDA levels and GSH-Px activity in Stattic-treated PANC-1 cells were not abolished by lentivirus-mediated STAT3 overexpression (Fig. S6D, E). Similarly, STAT3 overexpression did not affect GPX4 and xCT expression in PANC-1 cells treated with Stattic. Collectively, these results indicated that Stattic-induced ferroptosis may be cell-specific and has a certain connection with STAT3.

## Discussion

PC is a malignant tumor associated with high mortality. Abnormal cell proliferation caused by uncontrolled cell cycle drives PCC growth. Abnormal tumor cell cycles are caused by activated oncogenes. As a vital transcription factor, oncogene c-Myc is caused by damage to DNA and hypoxia [29]. Mutations in the TP53 gene occur in up to 70% of PC cases [30]. p53 acts to inhibit cell proliferation by transcriptionally activating target genes involved in regulating cell cycle, including genes producing cyclins and cyclin-dependent kinases (CDKs) [31]. Thus, c-Myc inhibition or p53 activation may potentially be utilized as a strategy against PC [29, 31]. This study showed that a non-peptide small molecule Stattic time- and dose-dependently inhibited PCC proliferation by reducing c-Myc expression and enhancing p53 activity. As a result, cyclin D1, p-Rb, Chk1, and p21 were suppressed in Stattic-treated PCCs. Cyclin D1, p-Rb, Chk1, and p21 are important regulators involved in the regulation of the G1 to S phases. In addition, decreased Ki67 expression and unchanged PCNA expression indicated Stattic-induced G1 cell cycle arrest. Furthermore, flow cytometry uncovered that PCCs were arrested at the G1 phase after Stattic treatment. Thus, Stattic effectively suppresses PCC proliferation by inducing cell cycle arrest at the G1 phase via downregulating c-Myc expression and upregulating p53 activity.

Apoptosis is a key mechanism of anti-tumor effect of Stattic. The caspases regulate apoptosis cascades [32]. Caspase activation induces cellular apoptosis dependent of two distinct but convergent pathways: the death receptor and mitochondrial pathways [33]. Caspase-3 is one of the most critical key executioners in the process of apoptosis and can cut many key proteins in the process of apoptosis such as PARP. The activation of caspase-3 requires cleavage from the inactive FL-Casp3 (35 kDa) between Asp28 and Ser29 and between Asp175 and Ser176 to generate active 17-kDa and 12-kDa subunits [34]. Here, Stattic upregulated CL-Casp3 expression, suggesting that PCC apoptosis was induced by Stattic. In addition, elevated Bax and cytochrome C expression and reduced bcl-2 expression indicated Stattic-induced mitochondrial-dependent apoptosis. Furthermore, cleaved-PARP and CL-Casp8 expression levels did not change, suggesting that Stattic-induced apoptosis may not involve the death receptor pathway. Interestingly, the mechanism of Stattic-triggered apoptosis for different tumor cells was different. In PANC-1 cells, Stattic induced apoptosis dependent on Bcl-2, whereas in BxPc-3 cells induced apoptosis by targeting survivin. Taken together, mitochondrial-dependent apoptosis was involved in Stattic-mediated anti-PC effects.

Ferroptosis, as a newly discovered and controllable method of cell death that is different from apoptosis and necrosis, has attracted much attention in recent years, and it has considerable potential in the treatment of cancer [24]. Ferroptosis may be related to the tumor suppressor gene TP53, but cancer cell lines with KRAS mutations cannot induce their ferroptosis well [25]. Our result uncovered that GPX4 expression in PANC-1 cells with KRAS mutations or in BxPc cells without KRAS mutations was slightly lower than that in PDECs, suggesting that PCCs have moderate ferroptosis regardless of the presence or absence of KRAS mutations, but this may be a response of PCCs to adapt to the hypoxic microenvironment [35]. Interestingly, Stattic did not change the expression of GPX4 and xCT in PANC-1 cells, but reduced their expression in BxPc cells, indicating that KRAS mutations may contribute to a certain extent to the pharmacological effect of Stattic on ferroptosis.

Targeted inhibition of the activation of key signals is one of the strategies for anti-tumor therapy [19]. STAT3 is considered to be one of the key signals driving tumor development [36]. In PC, STAT3 is activated by gene mutations (e.g., KRAS) or cytokines (e.g., IL-6), thereby inducing PCC proliferation and inhibiting apoptosis through upregulation of genes encoding cell cycle regulators and apoptosis inhibitors such as cyclin D1, c-Myc, and Bcl-2 [37]. In addition, abnormal activation of STAT3 signaling mediates the invasion and metastasis of PCCs through induction of epithelial-mesenchymal transition [38]. Here, we identified upregulated STAT3 activity in various PCCs. However, the activity of STAT3 markedly varied in different PCCs and may lead to different outcomes after treatment with the STAT3 inhibitor. We explored the impact of Stattic on STAT3 activity in a nude mouse tumorigenesis model and in two kinds of PCCs with different genetic backgrounds of KRAS/SMAD4 mutations. Previous studies have shown that Stattic inhibits STAT3 activity by antagonizing STAT3 phosphorylation, dimerization, and nuclear translocation [19, 20]. Our results showed that Stattic selectively inactivated STAT3 by inhibiting STAT3^Y705^ phosphorylation in PANC-1 cells, but not in BxPc-3 cells, suggesting the difference in anti-tumor mechanism of Stattic in different PCCs. The results of a nude mouse tumorigenesis model constructed using PANC-1 cells also confirmed the inhibitory effect of Stattic on STAT3^Y705^ phosphorylation. Other pathways such as WNT/β-catenin, mTOR, and Hedgehog/Gli signaling are involved in the occurrence and development of PC [39]. However, Stattic did not change the activities of these signaling pathways. Thus, inhibiting STAT3^Y705^ phosphorylation may be one of the main mechanisms of anti-PC of Stattic, which may be dependent on drug concentration.

Further studies also confirmed the inhibitory effect of Stattic on STAT3 activity. IL-6 was used to induce STAT3 activation, resulting in the abolishment of the anti-tumor effect of Stattic in PCCs. Interestingly, IL-6 reversed Stattic-mediated anti-proliferative effects, possibly by reducing c-Myc expression without affecting p53 activity. Furthermore, lentivirus-mediated overexpression of STAT3 partially reversed the anti-tumor effect of Stattic. However, surprisingly, contradictory to the effect of IL-6, STAT3 overexpression significantly reduced the activity of p53 in Stattic-treated PANC-1 cells, indicating a closer relationship between p53 and STAT3 than IL-6. Finally, IL-6 or STAT3 overexpression did not affect GPX4 and xCT expression in Stattic-treated PANC-1 cells, and thereby Stattic-induced ferroptosis may be cell-specific and has a specific connection with STAT3.

This study has a number of limitations. First, a genetic approach and an in vivo experiment involving the molecular mechanism of Stattic regulating STAT3 was not employed. In addition, the mode of action between Stattic and the JAK2/STAT3 pathway should be clarified. Importantly, the effectiveness and safety of Stattic in clinical anti-tumor research should be assessed.

In conclusion, Stattic time- and dose-dependently inhibited PCC proliferation by reducing c-Myc expression and enhancing p53 activity. As a result, cell cycle-related regulatory factors were downregulated and PCCs were arrested at the G1 phase. In addition, Stattic induced mitochondrial-dependent apoptosis and ferroptosis in specific PCCs. Further in vitro and in vivo studies showed that Stattic exerts its anti-tumor effect via suppressing STAT3^Y705^ phosphorylation and nuclear localization. IL-6 was used to activate STAT3 or lentivirus-mediated overexpression of STAT3 partially reversed Stattic-mediated anti-proliferation and pro-apoptotic effects of PCCs. Thus, Stattic exerts an anti-tumor effect via inhibition of STAT3^Y705^ phosphorylation and has potential clinical therapeutic value for PC.

## Materials and methods

### Cell culture and administration of drugs

Human pancreatic ductal epithelial cell (PDEC) line (hTERT-HPNE) and PCC lines PANC-1, CFPAC-1, BxPc-3, and Patu8988 were obtained from the Chinese Academy of Sciences Cell Bank (Shanghai, China). These were passaged in DMEM or Roswell Park Memorial Institute-1640 medium (RPMI-1640; Invitrogen, Grand Island, NY) supplemented with 100 μg/mL streptomycin, 100 U/mL penicillin, and 10% fetal bovine serum. BxPc-3 and PANC-1 cells were cultured in a 10-cm culture dish at a density of 1 × 10^6^ for 24 h. Upon reaching 70–80% confluency, BxPc-3 and PANC-1 cells were exposed to 4 or 10 μM Stattic (Fig. 1Ahttps://pubchem.ncbi.nlm.nih.gov/compound/2779853#section=2D-Structure, CAS No. 19983-44-9, MedChemExpress (MCE), Shanghai, China) with or without human recombinant IL-6 (MCE).

### Cell Counting Kit-8 (CCK-8) assay

A CCK-8 cell assay and cytotoxicity detection kit were used to assess the viability of PANC-1 and BxPc-3 cells. The experiment was conducted in accordance with the instructions provided by the manufacturer. Briefly, after resuspending the cells, 100 μL of cells were seeded in 96-well plates (containing 4 × 10^3^ cells per well) and incubated with 10 μL of the different concentrations of Stattic. This was followed by incubation with 10 μL of CCK-8 solution for 2 h, followed by absorbance reading using a microplate reader at a wavelength of 450 nm. This test was repeated thrice.

### Evaluation of effect on Stattic on colony formation

BxPc-3 and PANC-1 cells (containing 800 cells per well) were seeded in six-well plates for incubation with the corresponding Stattic concentration. After forming visible clusters, 4% paraformaldehyde (Solarbio, Beijing, China) was added to fix the cells, followed by staining with crystal violet (Solarbio, Beijing, China). The formed clones were directly visually counted.

### Immunofluorescence staining

Briefly, 4% paraformaldehyde was added to Stattic-treated PANC-1 and BxPc-3 cells for 30 min for fixation, and the cells were permeabilized with 0.1% Triton X-100 (Solarbio, Dalian, China) for 10 min, and then the cells were blocked with ready-to-use normal goat serum (BOSTER, Wuhan, China) to eliminate nonspecific fluorescence. After washing away goat serum, the cells were hybridized with primary antibodies (Table S1) at 4 °C for 24 h, and then with a fluorescent secondary antibody (DyLight 488-green or 594-red, ProteinTech, Wuhan, China). Finally, the cells were stained with DAPI (Solarbio, Dalian, China), and images were captured using a DM4000 B LED microscope system (Leica Microsystems, Wetzlar, Germany).

### Western blot experiment

This test was conducted as described in our previous report [40]. Briefly, total cellular proteins were purified from BxPc-3 and PANC-1 cells or PC tissues of nude mice that were subjected to western blotting using primary antibodies (Table S2) and horseradish peroxidase (HRP)-conjugated secondary antibody (Biyuntian Biotechnology Co., Ltd., Shanghai, China). Protein bands were quantified using Image-Pro Plus software (version 6.0) and normalized using GAPDH antibody (1:2000, Abcam, Shanghai, China).

### Assessment of cell cycle and apoptosis

The cell lines were treated with Stattic for 24 h and collected with EDTA-free trypsin solution, and then incubated with 5 μL of Annexin V-FITC reagent for 15 min at 4 °C in darkness. The cells were treated with 5 μL of 7-AAD Viability Staining Solution (Invitrogen, Grand Island, NY) for 5 min at 4 °C in the dark. After the adhered cell clumps were filtered, flow cytometry (Ex = 488 nm; Em = 530 nm, BD FACSVerse™, BD Biosciences, San Jose, CA, USA) was used for apoptosis analysis. Cell cycle analysis was the same as the cell pre-processing method for apoptosis analysis. First, the resuspended cells were incubated with cold PBS and cell fixation solution (70% ethanol). After fixing at −20 °C for 24 h, the ethanol was washed off, and the resuspended cells were treated with 500 μL of cold PBS. Second, the resuspended cells were incubated with 5 μL of RNase A Solution (10 mg/mL) (Solarbio) and 5 μL of 7-AAD Viability Staining Solution and then placed in water bath at 37 °C for 30 min and in the dark at 4 °C for 30 min. Finally, cell cycle was examined using flow cytometry. This test was repeated thrice.

### Nude mouse tumorigenicity experiment

Twenty-week-old male nude mice (BALB/c) weighing 20 ± 2 g were obtained from Wenzhou Medical University and housed under standard laboratory conditions. Before the experiment, 100 μL of PBS (containing 5 × 10^6^ PANC-1 cells) were subcutaneously administered to each nude mouse through the abdomen and back. After the tumor successfully constructed, each mouse was randomly assigned into two groups. Nude mice received daily injection consecutively for 4 weeks with Stattic treatment (10 mg/kg, *n* = 6) or solvent (DMSO and saline, *n* = 6). The weights of the nude mice were recorded one week after PCC injection. At the end of the experiment, the mice were sacrificed by anesthesia, after which tumor tissues were collected for subsequent experiments. The tumor volume was calculated (*V* = Shortest diameter^2^ × Longest diameter/2) [41]. In addition, the kidney, lung, spleen, heart, liver, and spleen were collected to assess Stattic toxicity. All mouse experiments were approved by the Laboratory Animal Centre, the First Affiliated Hospital of Wenzhou Medical University, China.

### Immunohistochemistry (IHC) and HE staining

Tumor tissues fixed in 4% tissue cell fixative (Solarbio, Dalian, China) were paraffinized and sliced into sections with 4-μm thickness. These were then dehydrated across an ethanol (100%, 95%, 85%, and 75%) gradient. This was followed by treatment with 25 μL of goat serum (BOSTER, Wuhan, China) to inhibit endogenous peroxidase and then heated for antigen retrieval in the presence of 0.1% sodium citrate buffer (pH 6.0). Next, we incubated the sections with primary antibodies (Table S1) overnight, followed by secondary antibodies (ProteinTech, Wuhan, China) with horseradish peroxidase for 1 h. The final analysis was performed after treating the sections with DAB Horseradish Peroxidase Color Kit. For HE staining, sections were stained in line with guidelines of hematoxylin and eosin kit (Solarbio, Dalian, China). The IHC and HE samples were semi-quantitatively or quantitatively evaluated by two independent researchers who were blinded to group allocation.

### Overexpression of STAT3

STAT3 overexpression in PANC-1 cells was induced by lentivirus infection. The lentivirus and negative controls were prepared by the Genechem (Shanghai, China). The lentivirus and 40 μL of virus infection reagent were added to the PANC-1 cells (1 × 10^5^) at a reinfection index (MOI) of 10 as per the manufacturer’s instructions. After overnight incubation, the medium containing the lentivirus was replaced with normal medium and further incubated for 48 h, and then 2 μg/mL puromycin was used to select puromycin-resistant cells. The main sequence of the lentivirus was Ubi-MCS-3FLAG-CBh-gcGFP-IRES-puromycin, and qRT-PCR, fluorescence staining, and western blotting assays were conducted to determine whether the cells were successfully induced to overexpress STAT3.

### qRT-PCR analysis

Total RNA was extracted from BxPc-3 and PANC-1 cells treated with or without Stattic using RNAiso Plus reagent (TaKaRa, Dalian, China), which was then used to prepare cDNA utilizing the PrimeScript RT reagent kit (Perfect Real Time) (TaKaRa, Dalian, China). After mixing cDNA with corresponding primers, the qRT-PCR reaction was run with SYBR Green reagent (Toyobo, Japan). All samples were analyzed based on ^ΔΔ^CT value method. The specific primers (Table S3) used were prepared by Sangon (Shanghai, China). In this test, β-actin was utilized as a reference.

### Statistical analysis

Statistical analysis was performed using GraphPad Prism software 8.0. Mean data between two groups were compared with two-sided Student’s *t*-test, whereas one-way ANOVA with Bonferroni’s post-test was used to compare multiple groups. The data were expressed as the mean ± standard error of the mean. *P* < 0.05 was set as the threshold of statistical significance.

## Supplementary information

Supplementary Text

Figure.S1

Figure.S2

Figure.S3

Figure.S4

Figure.S5

Figure.S6

Table
