# Supplementary material for: Inhibition of STAT3Y705 phosphorylation by Stattic suppresses proliferation and induces mitochondrial-dependent apoptosis in pancreatic cancer cells
Source: Cell Death Discov. 2022 Mar 14;8:116. doi: 10.1038/s41420-022-00922-9 (PMC8921333; doi:10.1038/s41420-022-00922-9)
Supplement: Supplementary file 1 — Supplementary Text [file 41420_2022_922_MOESM1_ESM.pdf]

Supplementary Text

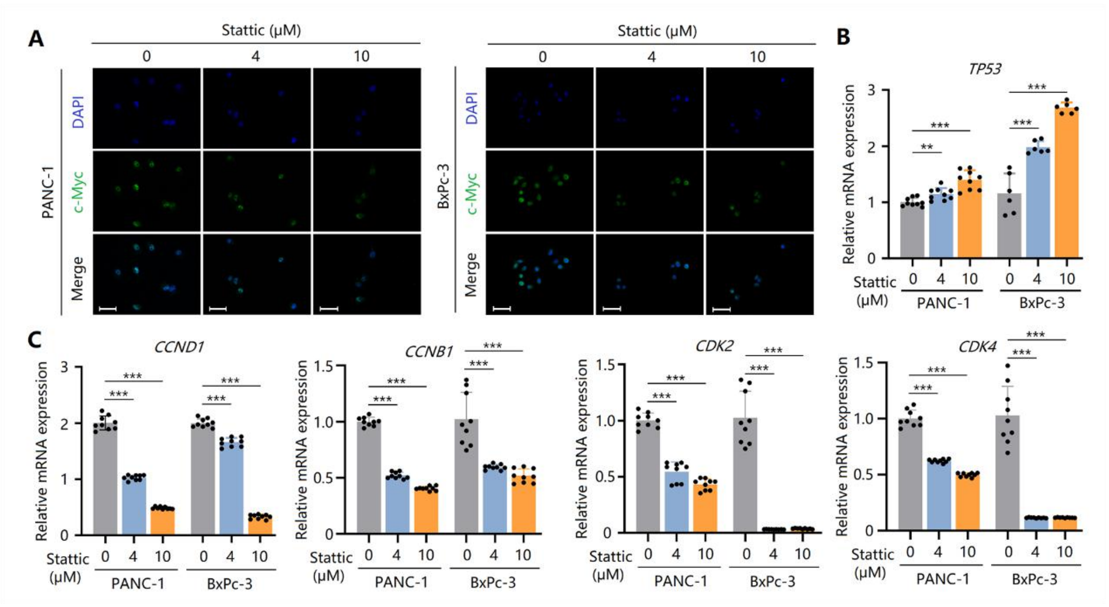

Fig. S1 Impact Stattic on cell cycle and proliferation of PCCs. A Results of immunocytochemical staining of c-Myc in indicated cells. Bar = 50 μm. **B** mRNA level of TP53 in the indicated groups. **c** mRNA level of CDK4, CDK2, CCNB1, and CCND1 in different treatments. $^{**}P < 0.01$, $^{***}P < 0.001$.

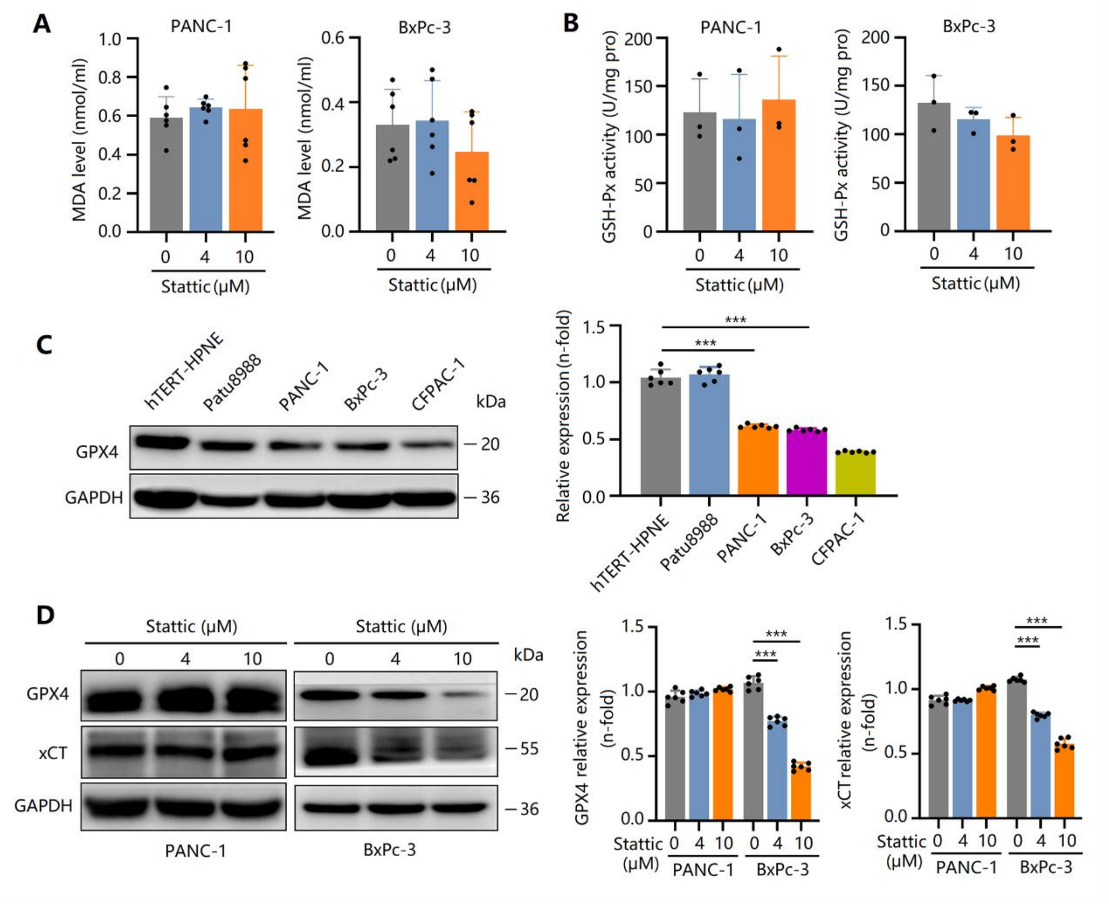

Fig. S2 Effects of Stattic on ferroptosis of PCCs. PCCs were treated with 4 and 10 μM of Stattic for 24 h. **A** Absorbance analysis showing the levels of MDA in Stattic-treated PANC-1 and BxPc-3 cells. **B** Absorbance analysis showing the activities of GSH-Px in Stattic-treated PANC-1 and BxPc-3 cells. **C** Western blot analysis showing the expression of GXP4 in different PCCs. **D** Expression of xCT and GXP4 in cells exposed to the indicated groups.

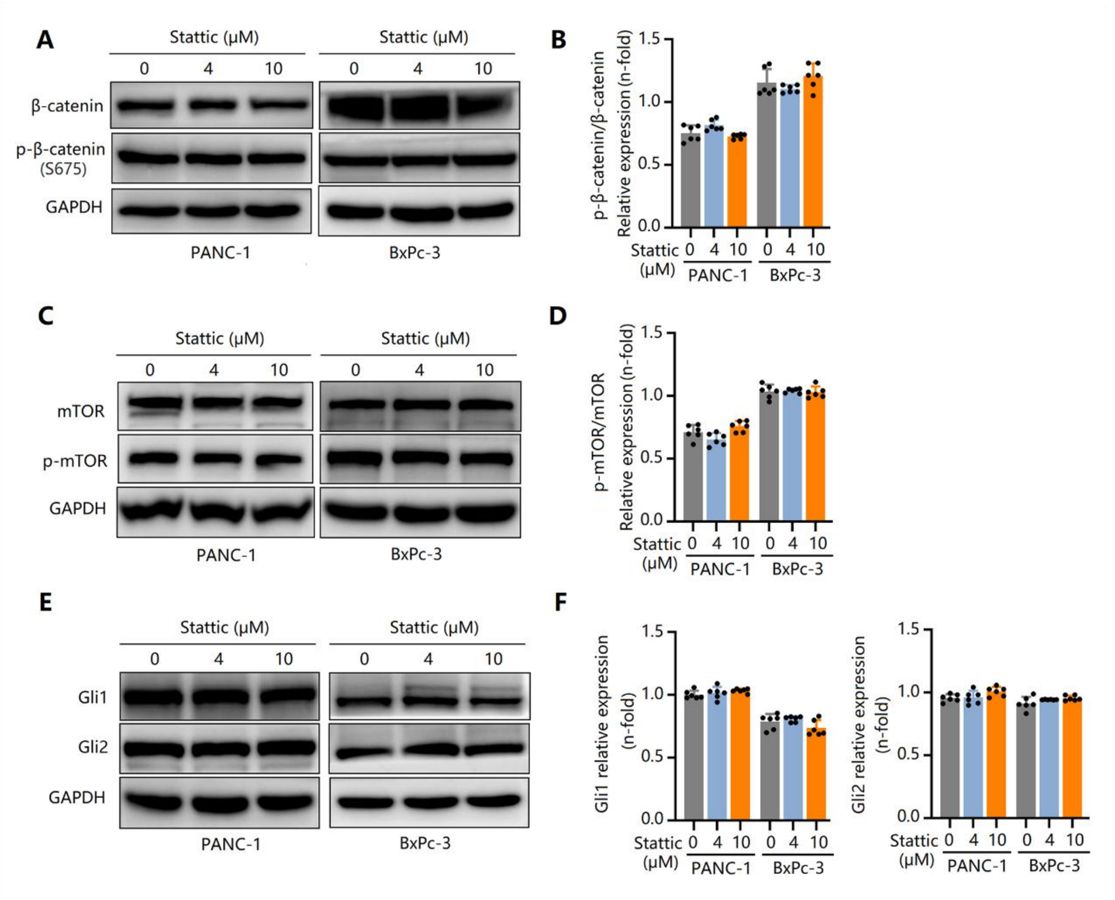

Fig. S3 Impact of Stattic on Hedgehog pathway, mTOR, and β-catenin. A, **B** Expression and phosphorylation of β-catenin in the cells under different treatment. **C**, **D** Expression level and phosphorylation of mTOR in cells from different groups. **E**, **F** Expression of Gli2 and Gli1 in in the indicated groups.

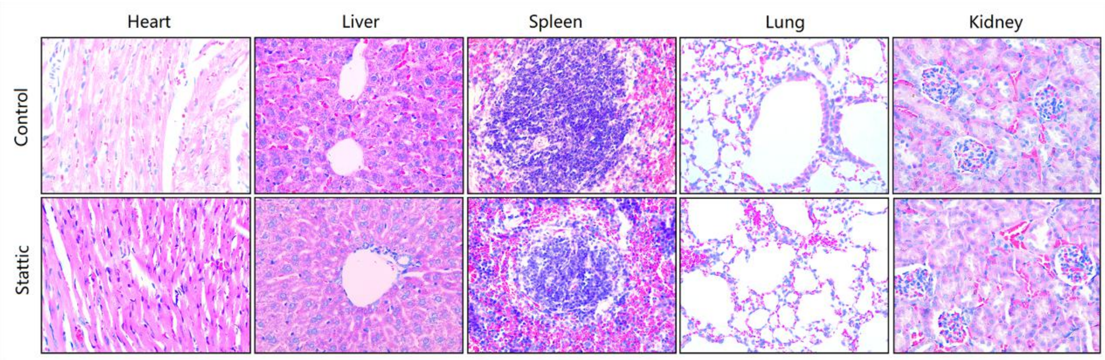

Fig. S4 Effects of Stattic on the tissues of heart, liver, spleen, lung, and kidney in nude mice. See Figure 6 for details of animal construction and drug treatment. HE staining was performed to evaluate the effects of Stattic on the tissues of heart, liver, spleen, lung, and kidney in nude mice. Bar = 100 μm.

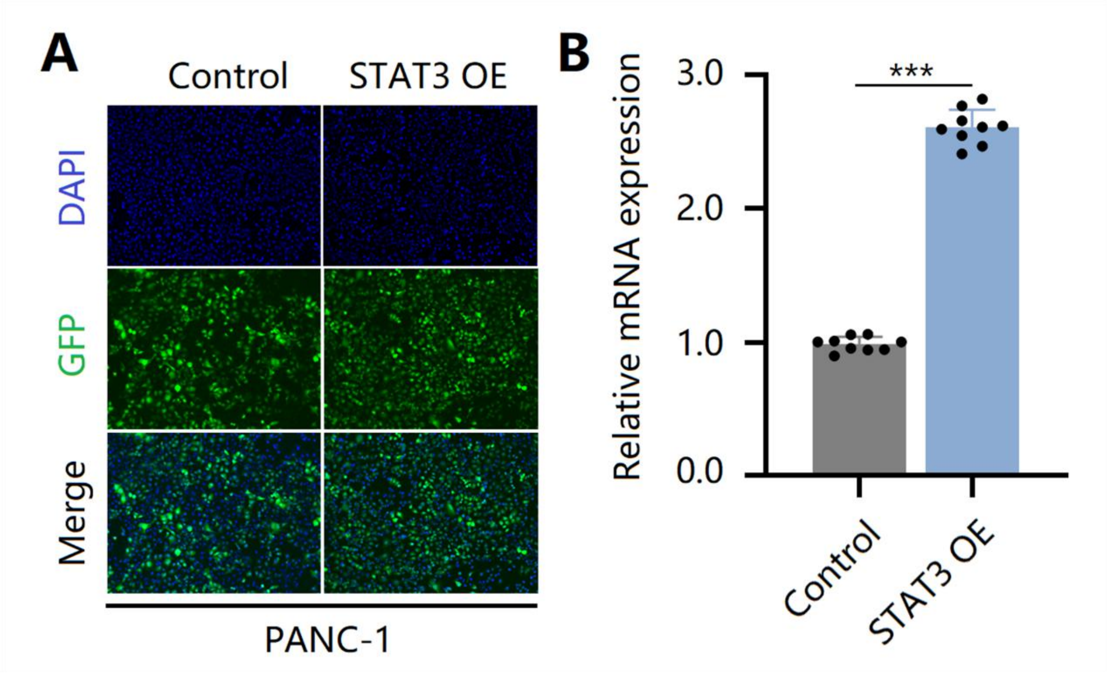

Fig. S5 Effects of lentivirus transfection on the expression of STAT3. A The transfection efficiency of lentivirus by immunofluorescence staining. **B** The mRNA expression of STAT3 in PANC-1 cells after transfection with lentivirus. Data were presented as the mean ± standard deviation in quintuplicate for the cell line experiment, and were analyzed by a two-sided Student's *t*-test. $^{***}P < 0.001$.

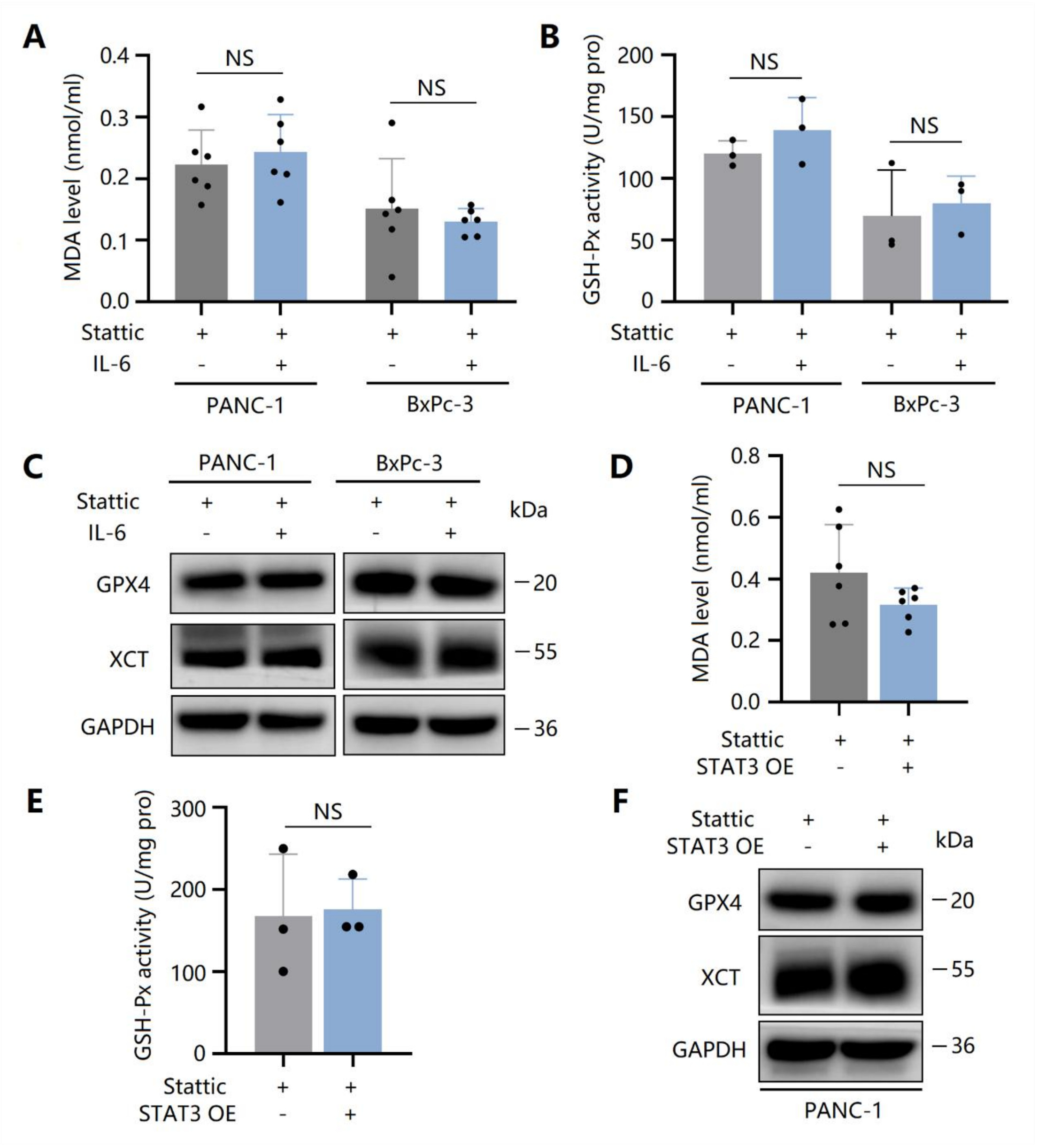

Fig. S6 Effects of IL-6 or STAT3 overexpression on ferroptosis of Stattic-treated PANC-1 cells. PANC-1 cells were treated with 10 μM of Stattic with or without STAT3 overexpression lentivirus or IL-6. **A** MDA levels in cells exposed to different treatments. **B** Activity of GSH-Px in in the respective groups. **C** Protein level of xCT and GXP4 in the indicated groups. **D** MDA level in various groups. **E** Activity of GSH-Px in the indicated treatment groups. **F** Expression of xCT and GXP4 in the indicated groups. NS, not significant.
