# Supplementary figures and images for: Inhibition of STAT3Y705 phosphorylation by Stattic suppresses proliferation and induces mitochondrial-dependent apoptosis in pancreatic cancer cells
Source: Cell Death Discov. 2022 Mar 14;8:116. doi: 10.1038/s41420-022-00922-9 (PMC8921333; doi:10.1038/s41420-022-00922-9)

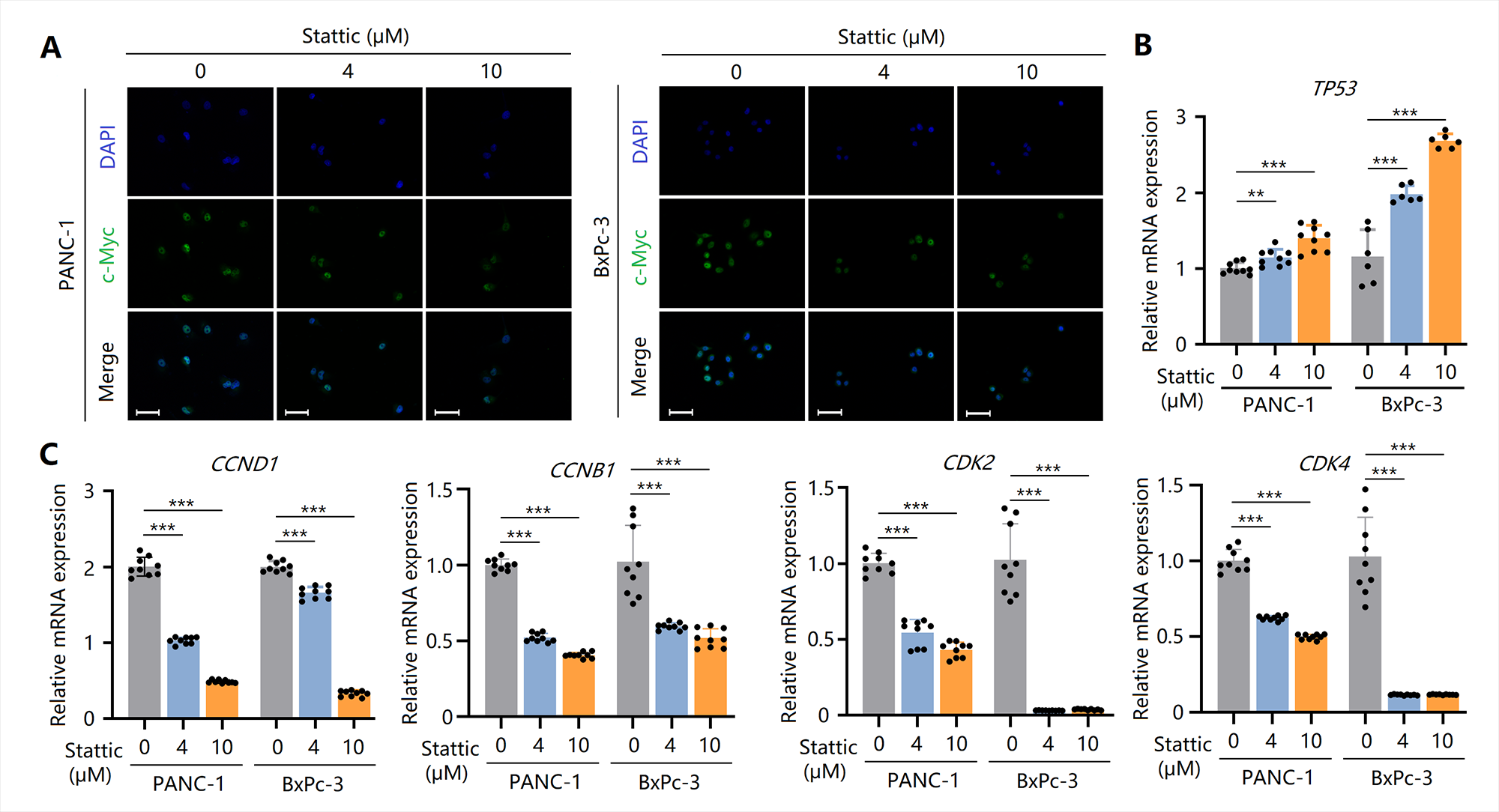

Supplement: Supplementary file 2 — Figure.S1 [file 41420_2022_922_MOESM2_ESM.tif]

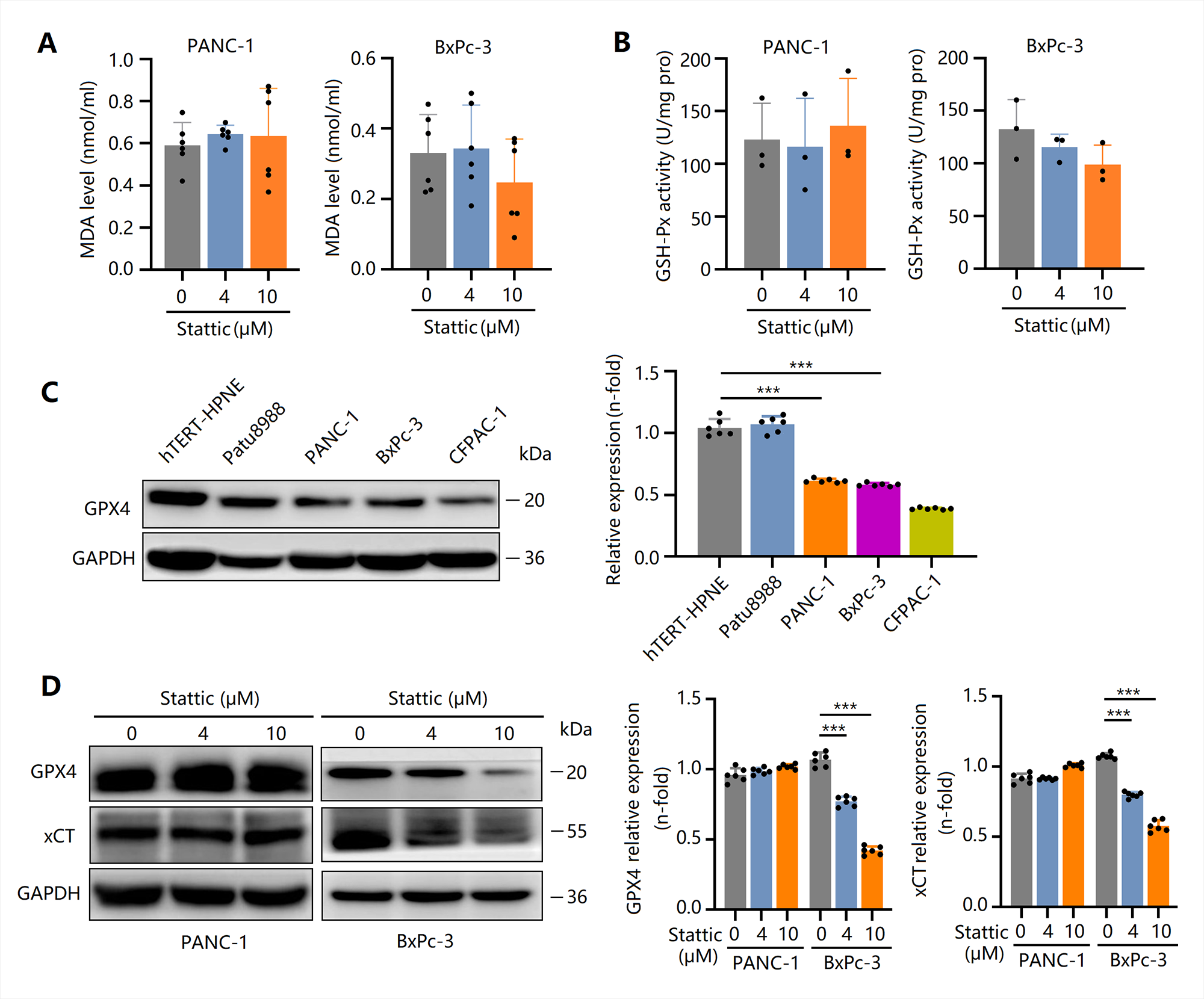

Supplement: Supplementary file 3 — Figure.S2 [file 41420_2022_922_MOESM3_ESM.tif]

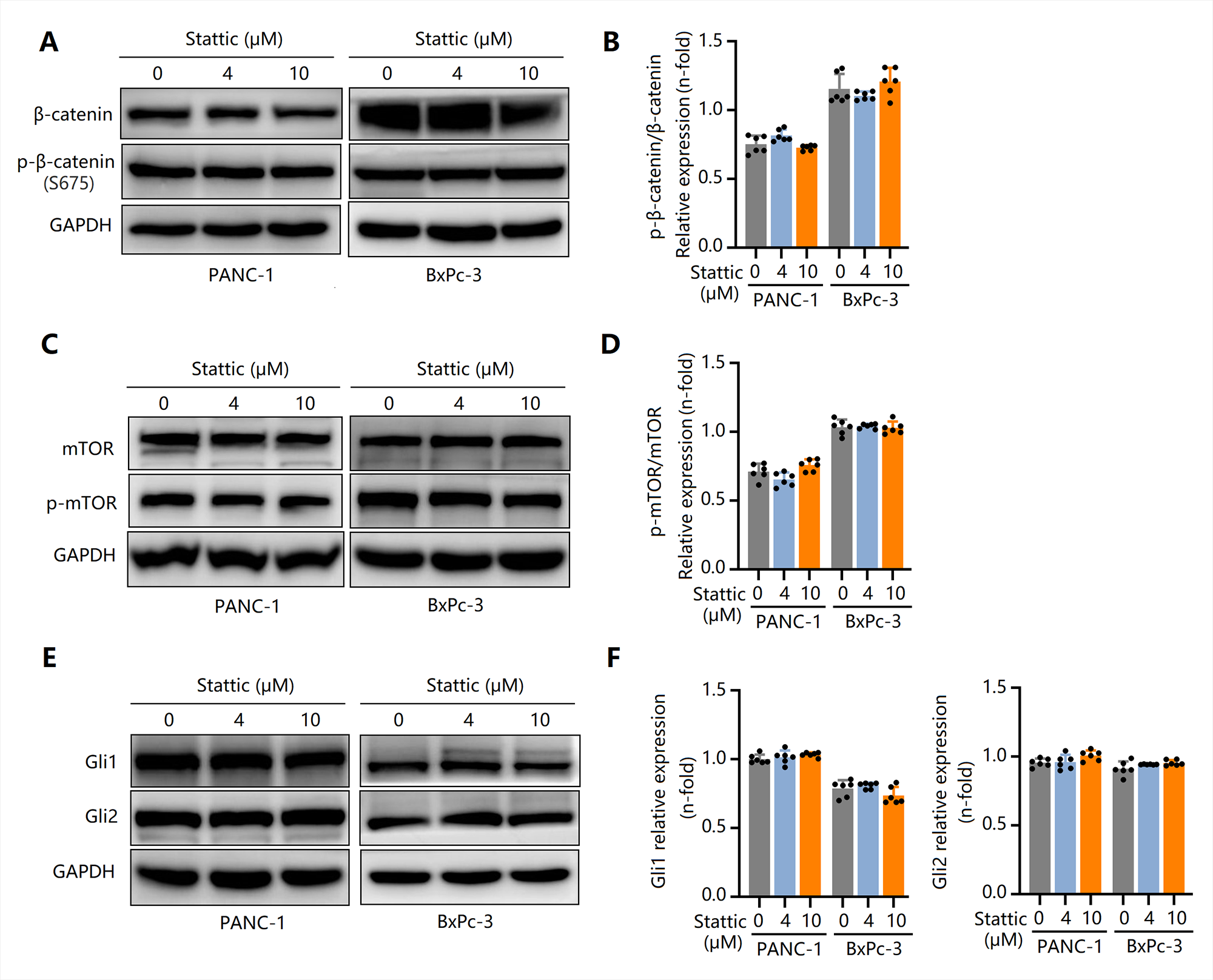

Supplement: Supplementary file 4 — Figure.S3 [file 41420_2022_922_MOESM4_ESM.tif]

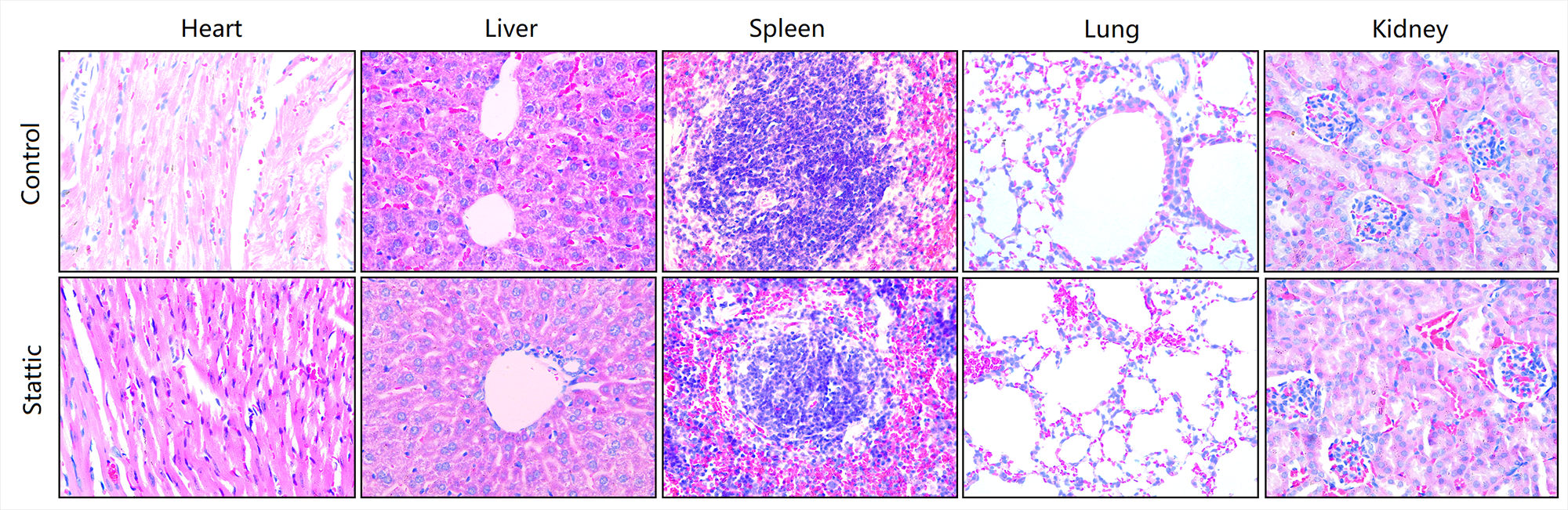

Supplement: Supplementary file 5 — Figure.S4 [file 41420_2022_922_MOESM5_ESM.tif]

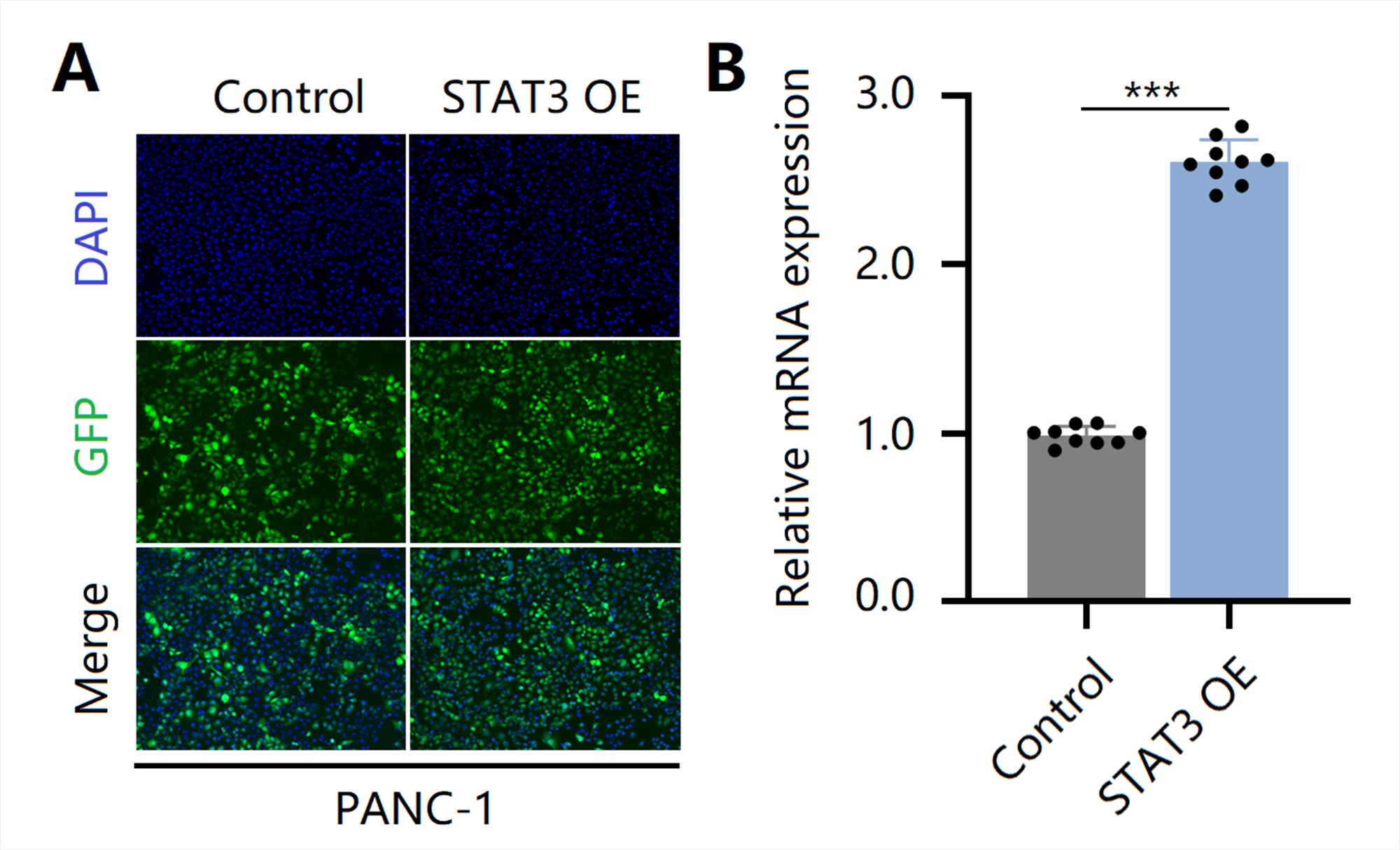

Supplement: Supplementary file 6 — Figure.S5 [file 41420_2022_922_MOESM6_ESM.tif]

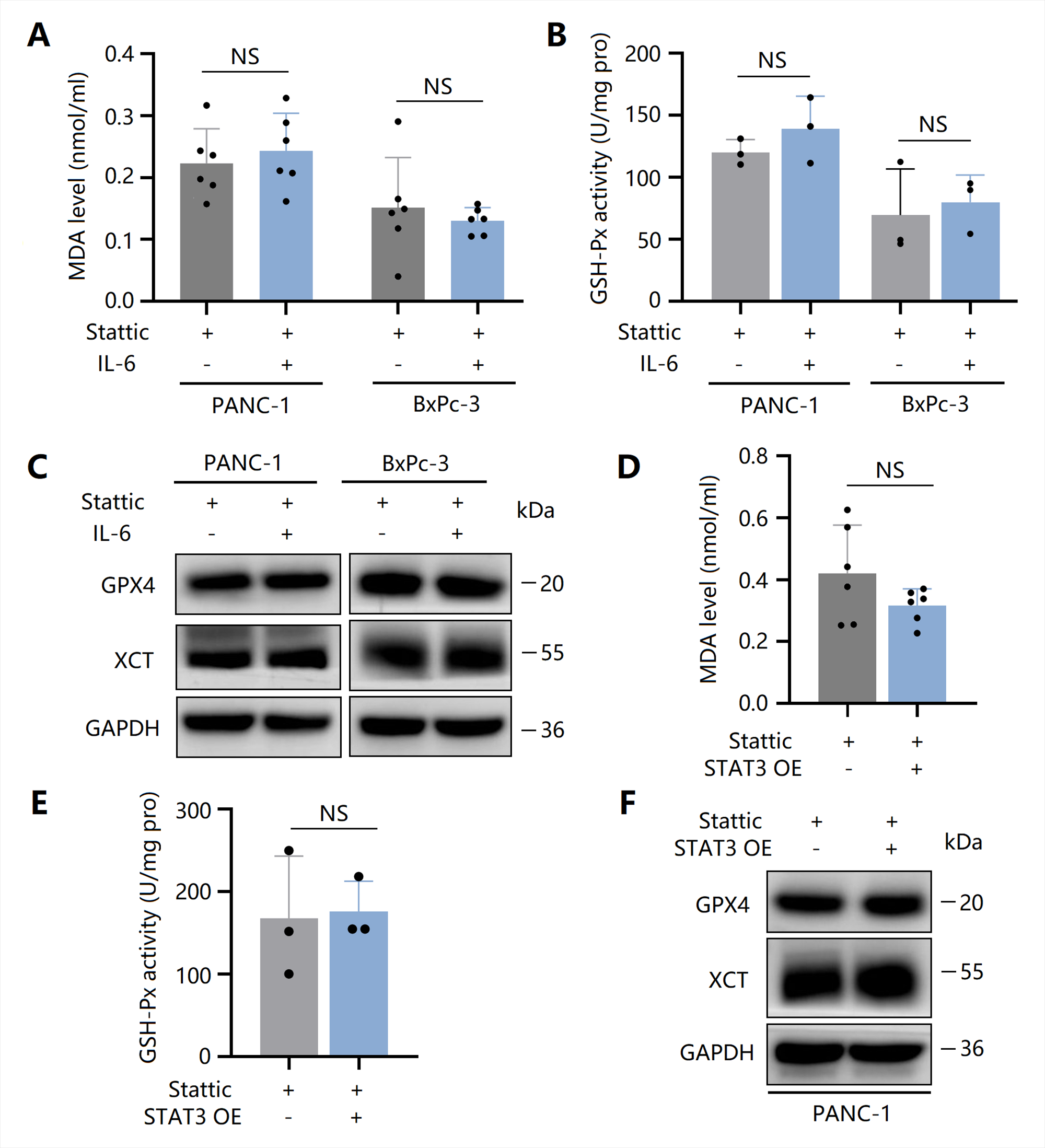

Supplement: Supplementary file 7 — Figure.S6 [file 41420_2022_922_MOESM7_ESM.tif]
