# Supplementary material for: Inhibition of STAT3Y705 phosphorylation by Stattic suppresses proliferation and induces mitochondrial-dependent apoptosis in pancreatic cancer cells
Source: Cell Death Discov. 2022 Mar 14;8:116. doi: 10.1038/s41420-022-00922-9 (PMC8921333; doi:10.1038/s41420-022-00922-9)
Supplement: Supplementary file 8 — Table [file 41420_2022_922_MOESM8_ESM.docx]

**Supplementary Table 1：The primary antibody information (IF and IHC of cells)**

| Antibody | Dilution ratio | Supplier |
| --- | --- | --- |
| p-STAT3 | 1:200 | Abcam, Shanghai, China, #ab76315 |
| p-JAK2 | 1:200 | Abcam, Shanghai, China, #ab32101 |
| Ki67  c-Myc  Cleaved-Caspase3  Bcl-2  Bax  Cytochrome C | 1:200  1:200  1:200  1:200  1:200  1:200 | Abcam, Shanghai, China, #ab16667  ProteinTech, Wuhan, China, #10828-1-AP  ProteinTech, Wuhan, China, #19677-1-AP  ProteinTech, Wuhan, China, #12789-1-AP  ProteinTech, Wuhan, China, #60267-1-Ig  ProteinTech, Wuhan, China, #10993-1-AP |

**Supplementary Table 2：The primary antibody information (Western blot analysis)**

| Antibody | Dilution ratio | Supplier |
| --- | --- | --- |
| JAK2 | 1:1000 | Abcam, Shanghai, China, #ab108596 |
| p-JAK2 | 1:1000 | Abcam, Shanghai, China, #ab32101 |
| STAT3  p-STAT3  c-Myc  Ki67（  PCNA  P53  p-P53  Cyclin D1  p-Cyclin D1  p-Rb  Chk1  P21  Cleaved-Caspase3  Cleaved-Caspase8  Bcl-2  Bax  Cytochrome C  Survivin  GPX4  xCT  GAPDH | 1:1000  1:1000  1:1000  1:1000  1:1000  1:1000  1:1000  1:1000  1:1000  1:1000  1:1000  1:1000  1:1000  1:1000  1:1000  1:1000  1:1000  1:1000  1:1000  1:1000  1:2000 | Abcam, Shanghai, China, #ab119352  Abcam, Shanghai, China, #ab76315  ProteinTech, Wuhan, China, #10828-1-AP  Abcam, Shanghai, China, #ab16667  Abcam, Shanghai, China, #ab92552  ProteinTech, Wuhan, China, #10442-1-AP  ProteinTech, Wuhan, China, #28961-1-AP  Cell Signaling Technology, MA, USA, #55506  Cell Signaling Technology, MA, USA, #3300  Abcam, Shanghai, China, #ab184796  ProteinTech, Wuhan, China, #25887-1-AP  ProteinTech, Wuhan, China, #10355-1-AP  ProteinTech, Wuhan, China, #19677-1-AP  ProteinTech, Wuhan, China, #13423-1-AP  ProteinTech, Wuhan, China, #12789-1-AP  ProteinTech, Wuhan, China, #60267-1-Ig  ProteinTech, Wuhan, China, #10993-1-AP  ProteinTech, Wuhan, China, #10508-1-AP  Abcam, Shanghai, China, #ab125066  Abcam, Shanghai, China, #ab175186  Abcam, Shanghai, China, #ab8245 |

**Supplementary Table 3：The sequence of specific primers**

| Gene name | Forward (5′-3′) | Reverse (5′-3′) |
| --- | --- | --- |
| β-actin  TP53  CCND1  CCNB1  CDK2  CDK4  STAT3 | CAACACAGTGCTGTCTGGCG  TCACCATCATCACTGGAAGACTC  CCTGTCCTACTACCGCCTCA  GATACTGCCTCTCCAAGCCCAATG  AGGATGTGACCAAGCCAGTACCC  TTGCCAGCCGAAACGATCAAGG  GAGGCAGGAGAATCGCTTGAACC | CGGAGTACTTGCGCTCAGGA  TTGGGCAGTGCTCGCTTAGT  TCCTCCTCTTCCTCCTCCTC  CAGCTCCATCTTCTGCATCCACATC  CCACCTGAGTCCAAATAGCCCAAG  TCCACCACTTGTCACCAGAATGTTC  TCTCAGACTGTCGCCCAGGATG |
